# Ptychographic Imaging of Branched Colloidal Nanocrystals Embedded in Free-Standing Thick Polystyrene Films

**DOI:** 10.1038/srep19397

**Published:** 2016-01-18

**Authors:** Liberato De Caro, Davide Altamura, Milena Arciniegas, Dritan Siliqi, Mee R. Kim, Teresa Sibillano, Liberato Manna, Cinzia Giannini

**Affiliations:** 1 Istituto di Cristallografia, Consiglio Nazionale delle Ricerche, via Amendola 122/O, 70126 Bari, Italy; 2 Istituto Italiano di Tecnologia (IIT), via Morego 30, IT-16163 Genova, Italy

## Abstract

Research on composite materials is facing, among others, the challenging task of incorporating nanocrystals, and their superstructures, in polymer matrices. Electron microscopy can typically image nanometre-scale structures embedded in thin polymer films, but not in films that are micron size thick. Here, X-ray Ptychography was used to visualize, with a resolution of a few tens of nanometers, how CdSe/CdS octapod-shaped nanocrystals self-assemble in polystyrene films of 24 ± 4 μm, providing a unique means for non-destructive investigation of nanoparticles distribution and organization in thick polymer films.

Hybrid materials fabricated by mixing metal chalcogenide semiconducting nanocrystals (NCs) with polymers have received particular attention in the last decade, triggered by their potential functionalities in the fields of electronics, optics, photovoltaics^1^,^2^,^3^,^4^,^5^, among others. The achievements in the synthesis of NCs in terms of control over their size and geometry^6^,^7^,^8^,^9^,^10^,^11^ have represented a step forward in the research of nanocomposites. As part of this field, scientists have been working on controlling the dispersion of NCs in polymer films and on correlating the formation of their ordered aggregates with the final properties of the nanocomposite^12^,^13^,^14^,^15^. The NCs organization in the polymer is expected to strongly influence the properties of the resulting material. For instance, the optical^16^, surface wettability^14^, stress sensing^17^ and transport^18^ properties of polymer nanocrystal-composites are strongly influenced by the NCs distribution and orientation in the polymer film. However, the presence of the polymer affects the way NCs interact during film fabrication, and additionally makes it difficult to directly visualize the NCs in the polymer, especially in μm-thick films, thus preventing the assessment of their distribution, orientation and formation of ordered aggregates. Therefore, a reliable non-destructive high resolution imaging technique with the capability to penetrate μm-thick samples and with the necessary resolution to visualize nanometre-scale structures is needed. This stringent requirement rules out electron-based microscopic techniques, as they are not suited for imaging μm-thick films. Hard X-rays on the other hand ensure full penetration in thick polymer foils (even when they are several tens of μm thick), but the non-periodic organization of the NCs in polymers requires a coherent X-ray beam, if a diffractive imaging approach is to be used.

In the last decade, Coherent Diffractive Imaging (CDI) has been developed and optimized for nanostructural analysis of complex materials, regardless of their degree of crystallinity, thanks to significant advances in synchrotron source brilliance, efficiency of focusing optics and detectors^19^. CDI has been developed as a very promising tool for materials investigation at the nanoscale. In this technique, a coherent X-ray beam, scattered by an ensemble of objects (without any spatial correlation), freely propagates until it reaches a 2D detector, where its diffraction pattern is registered^20^,^21^,^22^.

With respect to imaging techniques that exploit an image forming lens, CDI has the advantage of avoiding any objective lens which would limit the final image spatial resolution. In CDI the ultimate spatial resolution is determined, in principle, by the wavelength of the radiation. In reality, it is limited by the largest angle at which scattered light can be reliably detected, by the mechanical precision and stability of the entire setup, and by the sample radiation damage. As a drawback, CDI data have to be phase retrieved *a posteriori* with specific algorithms^23^,^24^,^25^ to reconstruct quantitatively the complex-valued exit wavefield after propagation through the object.

An interesting technique is ptychography, considered as the scanning mode version of CDI, which allows the analysis of extended specimens. In ptychography, the sample is scanned across a coherent and finite-sized illumination function-the probe-in such a way that the latter partially overlaps at adjacent scanning positions^26^,^27^,^28^. A diffraction pattern is recorded at each scanning position and it is then used in the phase retrieval algorithms to reconstruct the object transmission function^29^,^30^,^31^. The redundancy in the ptychography data, introduced by the overlapping illuminated regions, provides a higher robustness in the phase reconstruction algorithms. This technique has been employed with success in the last years, for example for the first tomographic reconstruction of a bone specimen^32^, as a label-free bioimaging method to investigate weakly scattering objects^33^,^34^ or biological cells^35^,^36^,^37^, as well as in Bragg projection for the visualization of strain fields of extended crystals^38^,^39^ or to image nanoscale ferroelectric domains^40^. The combination of ptychography with scanning X-ray fluorescence microscopy has also been explored^41^,^42^. Advantages of ptychography over the other X-ray based microscopy techniques can be listed as follows: i) it can probe matter at the nanoscale; ii) radiation damage problems are limited^33^,^34^,^35^,^36^,^37^, since it is a phase contrast imaging technique; iii) it is compatible with specific sample environments for *in situ* and *in operando* studies^38^,^39^,^40^; and iv) it can be combined with other types of microscopies^41^,^42^.

Here, ptychography was used to investigate the distribution of octapod-shaped NCs (made of a CdSe core and eight CdS arms)^10^,^43^ embedded in polystyrene (PS) μm-thick films. In nm-thick polymer films, octapods tended to either segregate to the bulk boundaries or to cluster into short linear arrays when the polymer content was increased, as revealed by transmission electron microscopy (TEM) in a previous work^14^. However, when the octapods were embedded in free-standing PS films, several μm thick, they could not be imaged through standard imaging techniques (such as TEM), which prevented us from drawing any conclusion on whether chain-like assemblies were still formed in polymers in such circumstances. Ptychography was therefore the technique of choice in this case. Phasing of the raw data, performed here using a difference map algorithm^27^, was combined with deblurring and denoising algorithms to improve signal-to-noise ratio and reduce the background contribution of the original data, and ultimately led to an enhancement of the object visibility. In this way, we were able to retrieve a high contrast octapod density map that enabled us to visualize the NCs organization in such thick polymer films.

## Results

**Figure 1:**
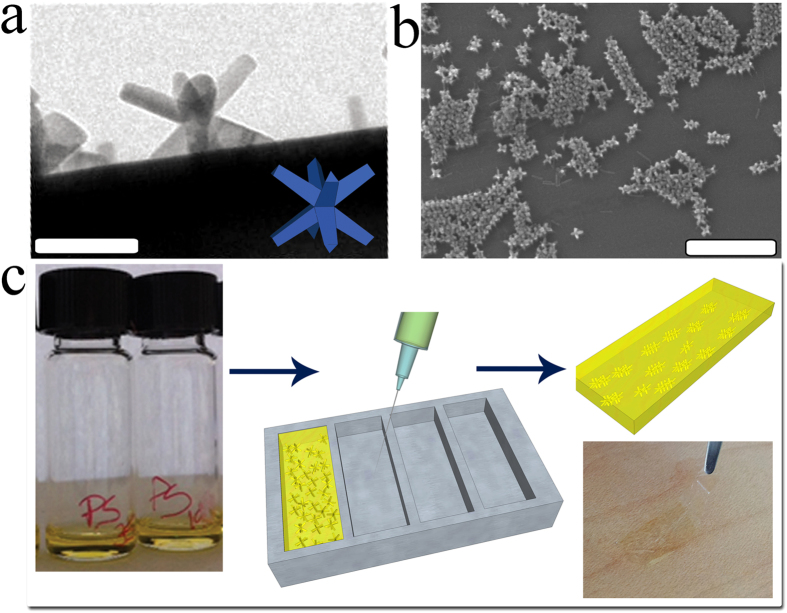
(**a**) Close-view TEM image showing a single octapod standing on a carbon-coated Cu grid with four pods. The cartoon highlights its orientation. Scale bar: 50 nm. (**b**) 45°-tilt-SEM image demonstrating the presence of a linear ordered aggregate of octapods after the removal of the PS by oxygen plasma in a thinner nanocomposite film standing on a SiO_2_ substrate. Scale bar: 500 nm. (**c**) Overview of the fabrication process by mould casting: vials containing the octapod solutions after the addition of the polymers evidencing no visible changes in transparency; the sketches show the injection of the nanocomposite solution into Al moulds to produce the free-standing thick films.

Figure 1 shows: a TEM image of a CdSe/CdS octapod-shaped NC synthesized as reported in previous works^10^,^43^ (panel a); the nanostructures formed by the addition of polystyrene (panel b); a scheme of the preparation process of the free-standing nanocomposite films for the ptycography studies (panel c). Details are given in the Supplementary Information (Fig. S1) and in the Methods section. The final thickness of the free-standing films was 24 ± 4 μm. For comparison purposes, additional samples from both repeatedly-washed octapod solutions and octapod/PS mixtures were also prepared by drop casting (15 μl) on Si_3_N_4_ membranes and were allowed to dry under the same conditions as for the free-standing films. The resulting nanocomposite films, prepared with this approach, presented a thickness of 307 ± 10 nm. The following specimens were structurally investigated: *i*) a sample made from a repeatedly-washed octapod solution (no polymer), drop-casted on top of a Si_3_N_4_ membrane (OCT sample); *ii*) two thin octapod/PS films, prepared with two different batches of PS having molecular weights (M_w_) of 190 and 350 kg/mol, respectively, and deposited on top of Si_3_N_4_ membranes (named PS190_thin and PS350_thin); *iii*) two free-standing octapod/PS films, named PS190 and PS350, using the same two polymer batches as for the films deposited on the Si_3_N_4_ membranes.

The first structural characterization was carried out by grazing incidence small angle X-ray scattering (GISAXS) on the named OCT sample and the thin octapod-PS films, PS190_thin and PS350_thin. Data were recorded at the XRD2 beamline of the LNLS synchrotron in Campinas. Fig. S2a–c are the GISAXS data registered on the OCT sample at incidence angles α_*i*_ of 0.17°, 0.27° and 0.37°, respectively. The coherent scattering features detectable in the data are fingerprints of the organization of the octapods in ordered arrays, as already reported by us in a previous work^44^. In order to better appreciate these features, GISAXS data collected on the OCT sample at α_*i*_ = 0.27° are displayed in Fig. S3 where the two dotted lines identify the position of intensity maxima associated to the presence of the arrays of octapods^44^. Conversely, GISAXS data collected, at the same incidence angles, from the PS190_thin and PS350_thin films (see Fig. S2d–i), did not reveal any scattering feature directly related to any possible assembly of the octapods into periodic arrays.

**Figure 2:**
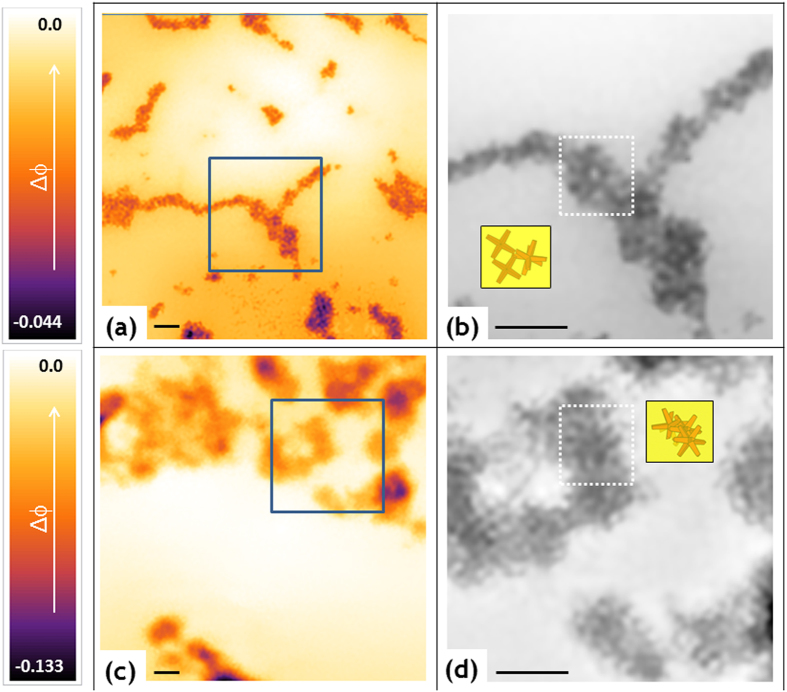
Ptychography results for the OCT (**a,b**) and PS350_thin (**c,d**) samples. Displayed in colors (**a,c**) and black and white (**b,d**) is the phase retardation (Δϕ) of the recovered complex-valued exit wavefield. Area of the images in panels (**a,c**) is 2.5×2.5 μm^2^, area of the images in panels (**b,d**) is 870×870 nm^2^ and corresponds to the square marked in blue in (**a,c**). Scale bar is 200 nm. Sketch in Fig. 2b depicts octapods in pod-to-pod contact visible in the square marked in white; sketch in Fig. 2d shows more intricate 3D aggregates with stronger interconnected octapods visible in the square marked in white.

**Figure 3:**
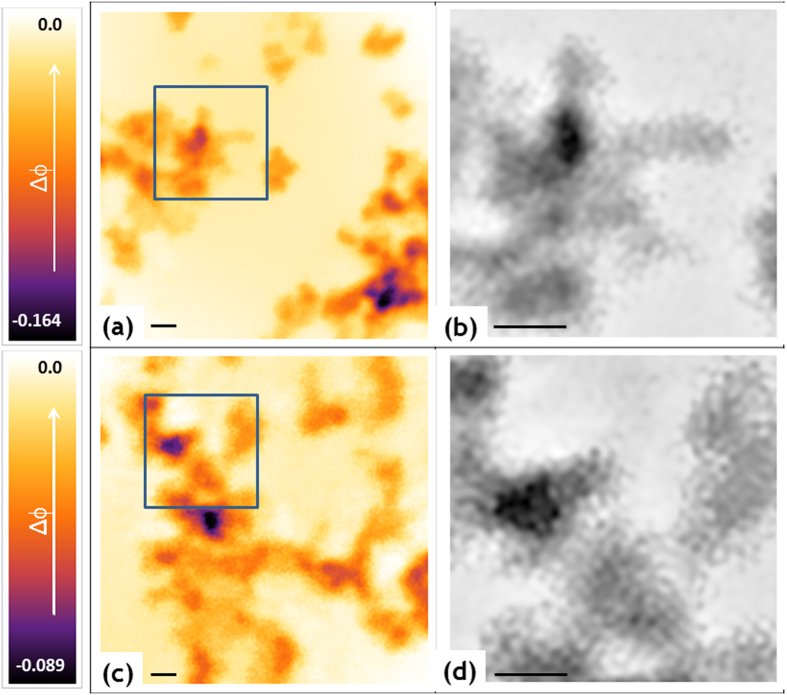
Ptychography results for the PS190 (**a,b**) and PS350 (**c,d**) free-standing samples. Displayed in colors (**a,c)** and black and white (**b,d**) is the phase retardation (Δϕ) of the recovered complex-valued exit wavefield. Area of the images in panels (**a,c**) is 2.5 × 2.5 μm^2^, area of the images in panels (**b,d**) is 870 × 870 nm^2^ and corresponds to the square marked in blue in (**a,c**). Scale bar is 200 nm.

PS350_thin, PS190 and PS350 free-standing films were therefore imaged through ptychography to inspect the configuration of the octapods in the PS thin/thick films, as a function of the polymer molecular weight M_w_ (comparison between the PS190 and PS350 films) and of the film thickness (comparison between the PS350_thin and PS350 films). The experiments, described in detail in the Methods section, were performed at the cSAXS beamline of the Swiss Light Source in Villigen. For the free-standing films (PS190 and PS350), two different areas (labelled as pos1 and pos2 in Table S1) distant 7 mm from each other were explored. A single area was investigated for the PS350_thin and the OCT samples. For each area we acquired data from five regions (named A, B, C, D, E as displayed in the scheme in Fig. S4). Each region was 4 × 4 μm^2^ large and the regions were located at relative distances of 50 μm from each other. The same region was scanned five times consecutively by repeating the procedure described in the Methods, producing a matrix of images 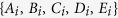, with *i* = 1 to 5. An example of the five phased maps acquired from sample PS190 in the region A of pos2 is shown in Fig. S5a–e. In all these images the structural features visibility is affected by noise and contrast. Consequently, averaging, denoising and deblurring were useful to reduce the noise level in the polymer background, to enhance the image contrast and therefore, to visualize the nanoscale structure of the sample at the experimentally achievable spatial resolution (see details in the Methods section). The image obtained after the averaging/denoising/deblurring process for sample PS190 is reported in Fig. S5f. Given the clear improvement that was observed, we proceeded to average, denoise and deblur all the phased images, after sub-pixel alignment. The results are presented in Fig. 2a for the OCT sample (region E), Fig. 2c for the PS350_thin (region A), and in Fig. 3a and Fig. 3c for the PS190 (region A, pos2) and PS350 (region B, pos2) free-standing films, respectively. These images represent the phase of the recovered complex-valued exit wavefield, after propagation through the object^25^ or, in other words, the images are correlated to the actual arrangement of the octapods inside the polymer thick-films. The full dynamical colour range was used in the images to enhance object visibility. The presence of the NCs produces an additional phase shift Δϕ with respect to the reference value corresponding to the bare polymer (background level). For each region and area, here explored, we computed and summarized in Table S1: *i*) the percentage (S%) of the 4 × 4 μm^2^ region where the phase Δϕ exceeds the background level, which is a direct indication of the sample portion containing nanocrystals; *ii*) the mean phase (<Δϕ>) averaged over S% and *iii*) the maximum phase retardation (Δϕ_max_). The error in the phase shifts is about 0.002.

To evidence the effect of the molecular weight and thickness of the polymer on the octapod arrangement we conducted a quantitative comparison among the four samples. In Table 1 we report the mean phase shifts <Δϕ>, extracted from Table S1, and the polymer thickness (t_PS_). We found that 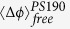 is larger than 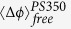 and 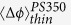
is larger than 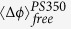. Thus, the average phase shift increases both by reducing the polymer thickness t_PS_, for the same molecular weight, and by reducing the polymer molecular weight, for the same t_PS_. As a result, the octapod aggregation appears to be dependent on the molecular weight and thickness of the polymer film.

Table 1 summarizes also the image resolution values (ρ). Considering the adopted experimental conditions described in the Methods section, the expected spatial resolution was ρ
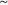
27 nm. In order to estimate the actual spatial resolution of the phased images (before and after the averaging and filtering procedure), we adopted the Fourier shell correlation (FSC) criterion, as described in the Supplementary Information (section Resolutions, Fig. S6)^45^,^46^. The error in the FSC resolution depends on the indetermination in the sub-pixel alignment procedure^46^. By changing the alignment by ±1 subpixel (Δ_det_/3), the resolution was found to vary within ±1 nm.

Close up views of the regions framed in blue in Figs 2 a,c and 3a,c, are displayed in Figs 2b,d and 3b,d, which allow to better elucidate the arrangement of octapods, both alone and embedded in a thick-free-standing polymer film. In the case of the octapods without polymer, the NCs are standing with four pods in contact with the Si_3_N_4_ membrane and forming linear arrays in some areas, where the octapods present a pod-to-pod contact with neighboring ones (as highlighted by the sketch in Fig. 2b which shows possible ways of NCs contact), while the octapods form more intricate 3D aggregates when they are embedded in the polymer films-with stronger interconnection-as depicted by the sketch in Fig. 2d. This can be also appreciated from the changes of the <Δϕ> values, that are the smallest in the case of octapods that form mainly 2D structures (see Fig. 2a,b and 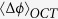 in Table 1) and they become larger when the octapods are embedded in the polymer, which is an indication of their 3D organization. Note that our images present features that cannot be exclusively attributed to the sample structures, as they can be residual artifacts of the reconstruction process, most probably due to a not entirely accurate knowledge of the probe translation positions^47^. However, the unavoidable presence of residual artifacts in the images does not impact significantly our conclusion of being able to visualize the NCs in the thick-polymer films. Thus, our results highlight the potential use of the ptycography for the analysis of nanostructures in polymer matrices.

## Conclusions

To summarize, the major results which can be drawn from this analysis are the following:

1. GISAXS investigation ruled out any possible organization of the octapods into ordered arrays for the thin polymer films; periodic arrays were found only for the sample made from a repeatedly-washed octapod solution (no polymer), drop-casted on top of a Si_3_N_4_ membrane.
2. Ptychography allowed visualizing the self-assembly of octapods into linear and interconnected structures, both in the thin polymer films and in the free-standing polymer films. This result is in agreement with the octapod configuration observed by TEM/SEM on nanometric thin polymer samples, but never experimentally demonstrated for free-standing thick films.
3. Averaging/deblurring/denoising allowed improving image contrast and reducing noise level in the background between octapod nanostructures. This consented to visualize the sample structures at a resolution close to the nominal one (27 nm).
4. Ptychographic data were used to explore the effect on the octapod aggregation of: *i*) different polymer film thickness for the same polymer molecular weight in the PS350_thin and PS350 samples; and *ii*) different molecular weights, for the same thickness of the polymer film in the PS350 and PS190 samples. The results from the analysis suggest that the octapod distribution in the polymer films is influenced by the molecular weight of the polymer and the thickness of the resulting composite film.
5. The resolution of the phase maps acquired in the free-standing composite films was not affected by the film thickness in the range here studied, up to 24 μm. This finding indirectly confirms that the projection and product approximations hold for our experiment. Actually, both film thickness and molecular weight do not impact the quality of the resulting images. This finding could have interesting implications for other imaging experiments and sample preparations.

In conclusion, we have imaged through ptychography the aggregation of CdSe/CdS octapod-shaped nanocrystals in 24 ± 4 μm thick free-standing polystyrene films. This X-ray-based microscopy technique allowed imaging, with a resolution of a few tens of nanometers, the aggregation of the octapods in interconnected architectures. Our work demonstrates the potential of ptychography to enhance the imaging of complex structures embedded in thick free-standing films, thus helping to fill the gap from nanometer to micrometer scale.

## Methods

### Preparation of the octapod-polystyrene nanocomposite films

The nanocomposite solutions were prepared by first dissolving polystyrene polymer from Sigma-Aldrich (M_w_ = 190.000 g/mol; M_w_ = 350.000 g/mol; T_g_ = 94 °C) at 5% vol in toluene; the polymer solutions were kept under strong shaking for 5 hours prior to use and then sonicated for 5 min. Octapods/polystyrene mixtures were prepared by adding 200 μl of sonicated polystyrene solutions to 300 μl of the sonicated octapod solution (10^−8^ M) for a final polymer concentration in the mixture of 2% vol. The mixtures were magnetically stirred rigorously for 15 min, and cleared of air bubbles. In the first series of experiments the mixture solution was cast on a SiO_2_ substrate. Then we proceeded to prepare the free standing films: 350 μl of the mixture prepared with the higher M_w_ polymer and and 450 μl of the mixture prepared with the lower M_w_ polymer were injected each mixture solution into an open Aluminum mould of 30 × 10 × 3 mm^3^ previously polished, cleaned and coated with Marbocote 227 CEE release agent from Marbo Italia SPA. The samples were allowed to dry at room temperature inside the glove box for 12 hours. Finally, the samples were released from the mould and cut in prismatic shapes of ca. 20 × 8 mm^2^.

### Bright field transmission electron microscopy (TEM) analyses

Bright field transmission electron microscopy (TEM) analyses were conducted on a 100 kV JEOL JEM 1011 microscope. Scanning electron microscopy (JEOL JSM-7500FA) was conducted on both carbon-coated film on TEM grids and SiO_2_ substrates with a 10 nm sputtered carbon coating. All substrates were cleaned before depositing the drops using 10 minutes of ultrasonic bath in acetone, distilled water and isopropanol. After cleaning they were carefully dried with pressurized air. A plasma reactor (Gambetti Tucano Multipurpose Plasma System) with an oxygen flow was used for the removal of the polymer from the SiO_2_ substrate. The plasma exposure time was 5 min at room temperature with a bias power level of 200 W. The thickness of the thinner films supported by the membranes was measured by an AMBiOS XP-2 Technology optical profilometer. A micrometer gauge was used to measure the thickness of the free-standing films.

### Grazing Incidence Small Angle X-ray Scattering Experiment

GISAXS measurements were performed at the XRD2 beamline at LNLS (Brazilian Synchrotron Light Laboratory) in Campinas (Brazil). A Pilatus 100 K detector was used, with 487 × 195 pixels, and 172 μm × 172 μm pixel size. The sample-to-detector distance was equal to 2.3 m; the radiation energy was E = 8.0 keV.

### Ptychographic Coherent Diffractive Imaging Experiment

The experiments were performed at the cSAXS beamline of the Swiss Light Source, at the Paul Scherrer Institut in Villigen, Switzerland. The energy of the incident beam was set to 6.2 keV (wavelength, λ = 0.2 nm). An Au Fresnel zone plate (FZP) with a diameter of 200 μm, fabricated on a 200 nm thick silicon nitride membrane, was used to define a coherent illumination onto the sample. The outermost zone width and the thickness of the FZP were 50 nm and 500 nm, respectively. The focal distance was 50 mm with a depth of focus of 50 μm. Coherent illumination of the entire lens aperture was ensured by using a horizontal aperture close to the source^48^. The sample was mounted on a piezoelectric stage allowing positioning with nanometer resolution in the two directions transversal to the beam propagation. Coherent diffraction patterns were recorded with a Pilatus 2M detector with Δ_det-pixel_ = 172 μm pixel size^49^ placed at z = 2.236 m from the sample with a flight tube in between, filled with He to reduce air scattering and absorption. Optics-sample distance was z_1_ = 270 μm.

We first obtained ptychographic images on a test specimen with large phase contrast features in order to obtain an initial probe for subsequent ptychographic imaging of the samples, as described for ptychography on weak scattering objects^34^. For the investigated samples, we placed the specimen at z_1_ = 270 μm from the focal plane of the lens, in such a way that the beam had a size of d = 450 nm at the sample position. For each sample we collected 5 repeated scans of the sample area. Each scan was realized by moving the sample along concentric circles^34^ with a radial step size of 0.2 μm and 5 points in the first circle. A diffraction pattern with 0.2 s exposure time was collected at each scanning point. The total scanned area was 4 × 4 μm^2^ with a total of 324 scanning points (see Fig. S7 in the Supplementary Information), which took about 2 minutes including the time required for sample positioning. Polymers are expected to suffer from radiation damage. Therefore, we preferred the strategy of acquiring 5 datasets, with 0.2 s exposure time per scanning point, in order to fractionate the dose among datasets. This choice allowed us to measure *a-posteriori* the correlation between consecutive phase maps and to average only those with the highest correlation (equal or larger than 97%), i.e. unaffected by radiation damage.

Ptychographic reconstructions were performed for each scan with 200 iterations of the difference map algorithm^50^ and 100 iterations of a maximum likelihood optimization algorithm used as a refinement^51^,^52^. In the ptychographic reconstructions we used the probe previously retrieved with the test specimen as the initial probe, which was then updated in each iteration^50^. The effective area of the detector used for phasing was N_det_ × N_det_ = 192 × 192 pixels, determining a pixel size of the phased image of 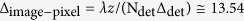 nm. The expected spatial resolution was 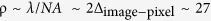 nm, being the numerical aperture 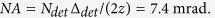


Concerning the procedure followed for denoising and deblurring the averaged images, image contrast enhancement was realized by means of a standard MATHEMATICA deblurring routine, based on the Total Variation approach, which deconvolves a point spread box function, one pixel large. Denoising was performed by a bilateral filter, which reduces noise, preventing edge smoothing.

## Additional Information

**How to cite this article**: De Caro, L. *et al*. Ptychographic Imaging of Branched Colloidal Nanocrystals Embedded in Free-Standing Thick Polystyrene Films. *Sci. Rep*. **6**, 19397; doi: 10.1038/srep19397 (2016).

## Supplementary Material

Supplementary Information

## Figures and Tables

**Table 1:** Mean phase retardation (<Δϕ>) and polymer thickness (t_PS_).

Sample	<Δϕ>	t_PS_ [μm]	ρ_original_[nm]	ρ_averaged_[nm]	ρ_averaged/filtered_[nm]
OCT	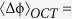 0.010±0.002	0	49.3 ±1.0	41.8 ±1.0	24.5 ±1.0
PS350_thin	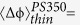 0.030±0.002	0.307 ±0.010	42.2 ±1.0	36.8 ±1.0	26.0 ±1.0
PS350	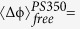 0.020±0.002	24 ± 4	52.5 ±1.0	39.4 ±1.0	32.5 ±1.0
PS190	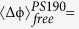 0.0275±0.002	24 ± 4	41.9 ±1.0	37.4 ±1.0	26.2 ±1.0

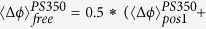
 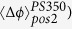. 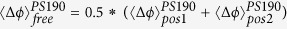, where 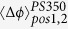 , 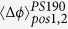 , 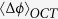, 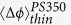
are reported in Table S1. Spatial resolution (ρ) on the original, averaged or averaged/filtered phased maps, as estimated by means of the FSC criterion (see Resolution section of the Supplementary Information).
