# Supplementary material for: Ptychographic Imaging of Branched Colloidal Nanocrystals Embedded in Free-Standing Thick Polystyrene Films
Source: Sci Rep. 2016 Jan 18;6:19397. doi: 10.1038/srep19397 (PMC4726119; doi:10.1038/srep19397)
Supplement: Supplementary Information [file srep19397-s1.doc]

**Supplementary Information**

**Ptychographic Imaging of Branched Colloidal Nanocrystals**

**Embedded in Free-Standing Thick Polystyrene Films**

Liberato De Caro^1^, Davide Altamura^1,#^, Milena Arciniegas^2,#^, Dritan Siliqi^1^, Mee R. Kim^2,§^, Teresa Sibillano^1^, Liberato Manna^2^ and Cinzia Giannini^1,*^

^1^Istituto di Cristallografia, Consiglio Nazionale delle Ricerche, via Amendola 122/O, 70126 Bari, Italy

^2^Istituto Italiano di Tecnologia (IIT), via Morego 30, IT-16163 Genova, Italy.

^§^Current Address: Institut National de la Recherche Scientifique (INRS), Université du Québec, 1650 Boulevard Lionel-Boulet, Varennes, Québec, J3X 1S2 Canada.

*correspondence and requests for materials should be addressed to [cinzia.giannini@ic.cnr.it](mailto:cinzia.giannini@ic.cnr.it)

^#^ these authors have equally contributed to the work

**Preparation of CdSe/CdS octapod-shaped nanocrystals**

*Chemicals:* Copper chloride (CuCl, 99.999%), tri-*n*-octylphosphine oxide (TOPO, 99%), *tri*-*n*-octylphosphine (TOP, 97%), and selenium (Se, 99.99%) were purchased from Strem Chemicals. *n*-Octadecylphosphonic acid (ODPA) and *n*-hexylphosphonic acid (HPA) were purchased from Polycarbon Industries. Propyl phosphoric acid (PPA), cadmium oxide (CdO, 99.99%), cadmium chloride (CdCl_2_, 99.99 %), sulfur (S, 99.98%), oleylamine (70%), 1-octadecene (90%) and chloroform were purchased from Sigma-Aldrich. Anhydrous methanol and toluene were purchased from Carlo Erba reagents. All chemicals were used as received.

**Synthesis of Cu_2-x_Se Seeds for Preparing Octapods**

All synthesis procedures described here were carried out using a standard Schlenk line. For the synthesis of Cu_2-x_Se seeds[^17^](#_ENREF_17) 1 mmol of CuCl was mixed with 5 mL of oleylamine and 5 mL of 1-octadecene in a 50 mL three-neck flask. The mixture was heated under vacuum at 80 ^o^C for 1 h and then heated up to 300 ^o^C under N_2_ flow. Meanwhile, a selenium precursor solution was prepared by dissolving 0.5 mmol of Se in 4 mL of oleylamine in a 25 mL three-neck flask. The mixture was put under vacuum at 130 ^o^C for 1 h and heated to 230 ^o^C under constant N_2_ flow. When Se was completely dissolved, the solution was cooled down to 180 ^o^C for a fast injection (by using a glass syringe equipped with a stainless steel needle) into the above copper solution kept at 300 °C. After injection, the reaction was run at 300 °C for another 15 minutes. The flask was then cooled down to room temperature and the resulting black solution was quickly transferred into a N_2_-filled vial for the sequent washing steps in a glove box under N_2_ atmosphere. Cu_2-x_Se nanocrystals were washed by repeated precipitation with methanol and re-dispersion in toluene, and the final seed nanocrystals dissolved in 3 mL of TOP. The nanocrystal concentrations of Cu_2-x_Se (with an average size of 15 nm) in TOP was determined to be around 3.0 x 10^-6^ M by inductively coupled plasma optical emission spectroscopy on solutions digested in a mixture of HCl:HNO_3_ (3:1, v/v).

**Synthesis of CdSe/CdS Octapods**

Briefly, 0.060 g of CdO, 0.006 g CdCl_2_, 0.290 g ODPA, and 3.000 g of TOPO were loaded in a 25 mL three-neck flask and heated to 120 ^o^C under vacuum for 1 h. The temperature of the mixture was switched to 380 ^o^C under N_2_ flow and 2.6 mL of TOP was injected. In the glove box, 100 μL of Cu_2-x_Se nanocrystals in TOP with a concentration of 3.0 **x** 10^-6^ M solution of (corresponding, therefore, to 3.0**·**10^-10^ moles of NCs) was mixed with 0.5 g of TOP:S (previously prepared by dissolving 96 mg of S in 1 mL of TOP). The mixture was then injected rapidly into the reaction flask recovered to 380 °C. After the injection, the reaction was run for 10 min before cooling to room temperature. The resulting product was purified several times in order to remove the excess of surfactant deriving from the synthesis and ensure a more direct contact with polymers. This process consisted of repetitive washing of the octapod solution with toluene and methanol, followed by heating at 70°C for 5 min and centrifugation at 3000 rpm for 3 min. The final precipitated was dispersed in chloroform and sonicated for 10 min. Figure S1a presents a TEM image of the solution with octapods after solvent evaporation at room temperature, showing a top view of the octapods standing on four pods in contact with the substrate and four pods coming out, forming a cross-shape view. By the TEM image analysis, the octapods presented a pod length, *L* of 39.1 ± 2 nm and pod diameter, *D_p_* of 12 ± 2 nm (see Figure 1a). Dynamic Light Scattering (DLS) measurements of the octapods in chloroform resulted in a narrow distribution with an effective particle size of 89.64 ± 0.5 nm (see Figure S1b) confirming that the nanocrystals were well dispersed in the chloroform solution before mixing with the polymer solutions, without evidencing the presence of aggregates.


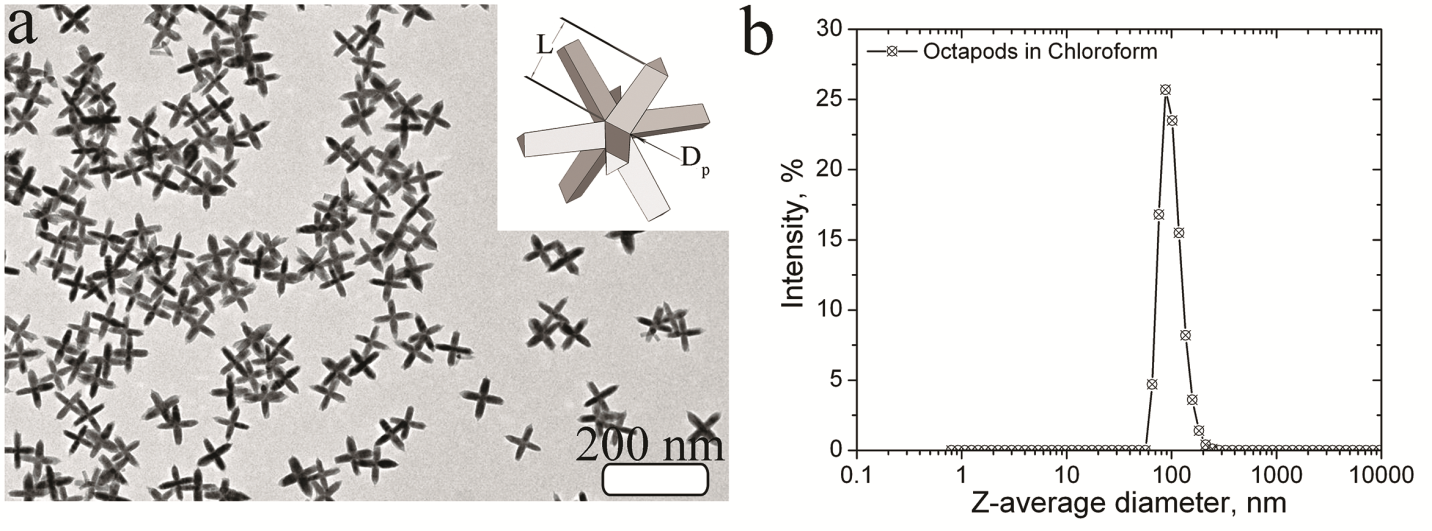


***Figure S1.*** *a) TEM image of the octapods after solvent evaporation. The inset shows a model of one octapod with a pod length, L and a pod diameter D_p_. b) Size-distribution profile obtained by DLS of the octapods in chloroform confirming that the octapods remained monodispersed and did not aggregate in solution. The log scale for the z-average diameter helps to evidence that the solution did not contain population sizes of different order of magnitude as aggregates.*

**GISAXS data**

GISAXS data recorded on the OCT (Fig. S2a-c and Fig. S3) sample, PS190_thin (Fig. S2d-f) and PS350_thin (Fig. S2g-i) films at the XRD2 beamline of the LNLS synchrotron in Campinas.

**
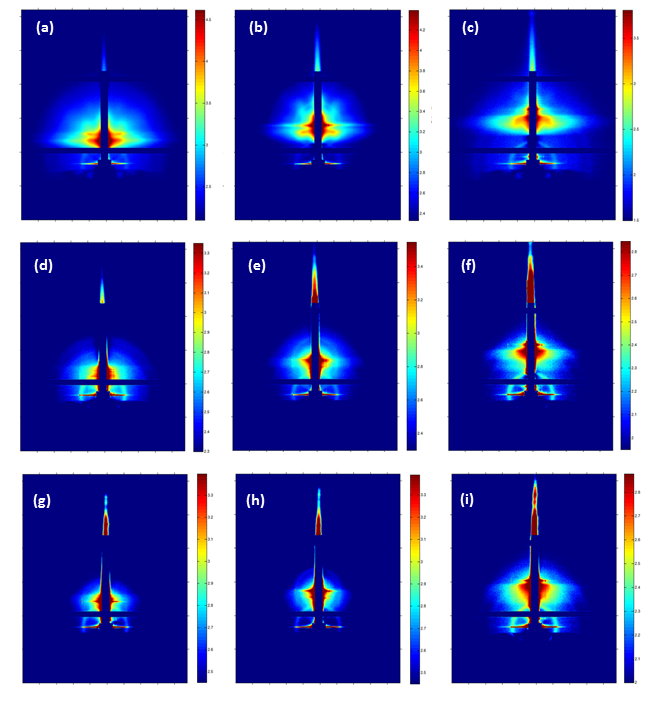
**

***Figure S2.*** *GISAXS data registered on the OCT sample (a,b,c), on the PS190_thin (d,e,f) and PS350_thin (g,h,i) films at incidence angles: α_i_ = 0.17° (a,d,g), α_i_ = 0.27° (b,e,h) and α_i_ = 0.37° (c,f,i).*

**
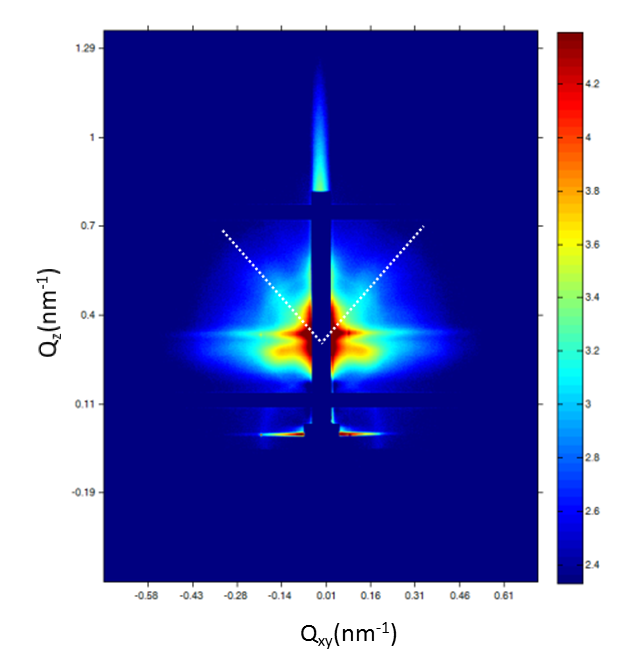
**

***Figure S3.*** *GISAXS data registered on the OCT sample at α_i_ = 0.27°. The white dotted lines, oriented at ~75 ° each other, are added to mark two directions along which intensity maxima are clearly detectable.*

**Ptychographic data**

**Statistical analysis**

D

C

E

A

B

Investigated regions

50 μm distance

***Fig. S4.*** *Scheme illustrating the five regions scanned from each area. The regions are distant approx. 50 µm from each other* *(drawing not in scale).*

| **Sample - Position** |  | **S%**  **over the**  **4×4 μm^2^ area** | **Average phase retardation (<Δφ>) over the coverage area** | **Maximum**  **phase**  **retardation (Δφ_max_)** |
| --- | --- | --- | --- | --- |
| PS190 – pos1 | A | 8.2 ± 0.5 | 0.036 | 0.119 |
| PS190 – pos1 | B | 10.8 ± 1.1 | 0.037 | 0.071 |
| PS190 – pos1 | C | 9.9 ± 0.7 | 0.024 | 0.103 |
| PS190 – pos1 | D | 8.7 ± 0.5 | 0.021 | 0.071 |
| PS190 – pos1 | E | 7.0 ± 0.6 | 0.021 | 0.091 |
|  | **AvePS190 – pos1** | **=8.9 ± 0.5** | 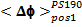**=0.028** | 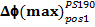**=0.091** |
| PS190 – pos2 | A | 34.3 ± 1.7 | 0.034 | 0.164 |
| PS190 – pos2 | B | 21.2 ± 1.1 | 0.022 | 0.114 |
| PS190 – pos2 | C | 21.9 ± 1.3 | 0.026 | 0.091 |
| PS190 – pos2 | D | 19.4 ± 1.1 | 0.026 | 0.106 |
| PS190 – pos2 | E | 18.6 ± 1.1 | 0.026 | 0.095 |
|  | **AvePS190 – pos2** | 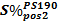**=20.0± 1.0** | 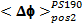**=0.027** | 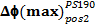**=0.114** |
| PS350 – pos1 | A | 8.3 ± 0.6 | 0.018 | 0.069 |
| PS350 – pos1 | B | 7.3 ± 0.7 | 0.020 | 0.077 |
| PS350 – pos1 | C | 11.8 ± 0.9 | 0.021 | 0.096 |
| PS350 – pos1 | D | 8.1 ± 1.1 | 0.018 | 0.078 |
| PS350 – pos1 | E | 10.4 ± 0.9 | 0.017 | 0.069 |
|  | **AvePS350 – pos1** | 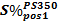**=9.0 ± 1.0** | 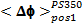**=0.019** | 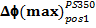**=0.078** |
| PS350 – pos2 | A | 17.1 ±1.5 | 0.023 | 0.135 |
| PS350 – pos2 | B | 40.9 ±2.2 | 0.022 | 0.089 |
| PS350 – pos2 | C | 29.3 ±3.2 | 0.024 | 0.093 |
| PS350 – pos2 | D | 29.1 ±3.3 | 0.019 | 0.078 |
| PS350 – pos2 | E | 22.9 ±2.9 | 0.018 | 0.095 |
|  | **AvePS350 - pos2** | 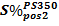**=32.0± 7.0** | 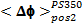**=0.021** | 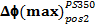**=0.098** |
| PS350 – thin | A | 14.0 ±1.5 | 0.039 | 0.140 |
| PS350 – thin | B | 11.2 ±1.2 | 0.038 | 0.230 |
| PS350 – thin | C | 10.0 ±1.2 | 0.032 | 0.140 |
| PS350 – thin | D | 8.2 ±2.3 | 0.032 | 0.087 |
| PS350 – thin | E | 15.0 ±2.9 | 0.048 | 0.180 |
|  | **AvePS350-thin** | **=11.7 ± 1.5** | 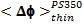**=0.030** | 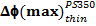**=0.155** |
| OCT | A | 8.0 ±1.3 | 0.009 | 0.03 |
| OCT | B | 6.0 ±1.0 | 0.010 | 0.08 |
| OCT | C | 11.0 ±1.5 | 0.010 | 0.03 |
| OCT | D | 15.0 ±2.0 | 0.010 | 0.06 |
| OCT | E | 15.0 ±2.5 | 0.010 | 0.05 |
|  | **AveOCT** | ***S%_OCT_*=11.0 ± 2.2** | **<Δφ>_OCT_=0.010** | **Δφ_OCT_ (max)=0.05** |

***Table S1.*** *Coverage percent (S%), average phase retardation (<*Δφ*>) and maximum phase retardation (*Δφ_max_*) values calculated for each region in the samples.*

***Figure S5.*** Original phased images (panels a-e), recorded for sample PS190, compared with the averaged/deblurred/denoised image (panel f)*.*

**
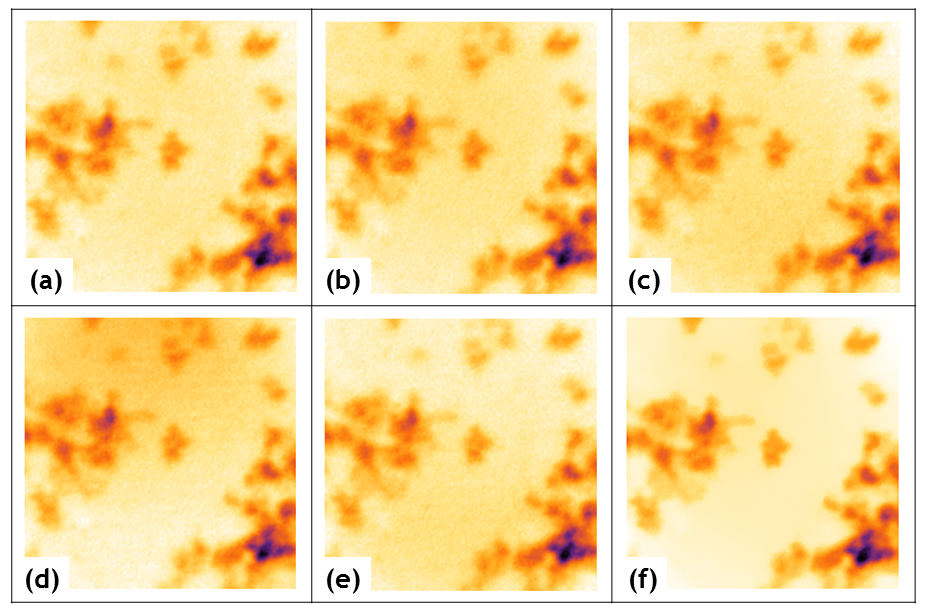
**

**Projection Approximation**

Standard Ptychography phasing stands on precise assumptions on the X-ray coherent lengths of the impinging radiation, as well as on the product and projection approximations. It is worth proving that, at a given spatial resolution, for the thick samples investigated here, these approximations are still satisfied, as discussed here.

It is well known that the permittivity ε of all materials in the X-ray range only slightly differs from its free-space value |ε-1|<<1. Physically, this causes that all diffraction processes in non-periodic samples have unidirectional and almost paraxial character. This property leads to the possibility to describe the perturbation of the sample on the incident wavefield by means of a two-dimensional (2D) *T*(*x*,*y*) function, known as “projection approximation”. The latter corresponds to the limit of geometrical optics where the propagation of light inside the sample can be described along straight rays and diffraction effects can be neglected. Indeed, the condition |ε-1|<<1 allows writing the wave equation in the parabolic approximation^1^, neglecting the second derivative $\partial^{2}u/\partial z^{2}$ of the slowly-varying propagation amplitude *u,* as:

$2ik\frac{\partial u}{\partial z}+\nabla^{2}u+k^{2}\alpha\left(x,y,z\right)u=0$, (S1)

where α = ε − 1 and *k* = 2π ⁄ λ. When the transversal diffusion term $\nabla^{2}u$ of equation (1) (Laplacian with respect to the *x* and *y* coordinates) is negligible with respect to the propagation term $2ik\partial u/\partial z$ we have

$2ik\frac{\partial u}{\partial z}+k^{2}\alpha\left(x,y,z\right)u=0$ (S2)

which, by direct integration, leads to the projection approximation

$u\left(x,y,t\right)=u\left(x,y,0\right)\exp\left[ik\int_{0}^{t} \frac{\alpha\left(x,y,z\right)}{2}dz\right]\equiv T(x,y)u(x,y,0)$, (S3)

being *T*(x,y) the 2D complex transmission function describing the action of an object on the incident wavefield.

In order to estimate in what conditions the projection approximation is valid one needs to compare the transversal diffusion term $\nabla^{2}u$ of equation (S1) with the propagation term $2ik\partial u/\partial z$. If we denote with *a* the characteristic scale of transversal non-uniformity of the object dielectric susceptibility, and with *b* the longitudinal one, the terms $\nabla^{2}u$ and $2ik\partial u/\partial z$ will be, respectively, of order ^2,3^:

$\nabla^{2}u\approx\frac{\Delta u}{\left(a/2\right)^{2}}$, (S4)

$2k\frac{\partial u}{\partial z}\approx\frac{4\pi\Delta u}{\lambda b}$. (S5)

The factor 2 in Eq. (S4) is due to the fact that the wave amplitude *u* goes from its minimum to the maximum at half of the transversal characteristic scale *a*. When the Fresnel parameter

$f=2k\frac{\partial u}{\partial z}/\nabla^{2}u\approx\frac{\pi a^{2}}{\lambda b}$ (S6)

is greater than 1, the projection approximation can be applied (*f* > 1).

By putting *a* ~ ρ and *b* ~ *t,* the validity of the projection approximation requires that the first Fresnel zone (λ*t*)^0.5^*,* for waves emitted by a point and propagated along a distance *t*, has to be much smaller than a circular area of radius ρ:

$t<\frac{\pi\rho^{2}}{\lambda}$. (S7)

In the present cases, for ρ = 40 ÷ 50 nm and λ=2Å, Eq. (S7) gives *t* < 25µm and, therefore, this approximation seems to be almost completely satisfied. However, it is worth noting that *b* ~ *t* would mean that the total thickness of the polymer is filled with chains of nanocrystals, even aligned along its thickness, the latter condition being highly different from the presented cases where nanocrystal chains are quite diluted in the matrix. In other words, in the experimental cases under study, the characteristic scale *b* of longitudinal non-uniformity of the object dielectric susceptibility is much smaller than the sample thickness *t* and, consequently, the projection approximation is fully satisfied.

Another approximation whose validity needs to be verified is the “product approximation”. Ptychography phase reconstructions are usually based on the above approximation, because the exit wave *u*(*x*,*y*,t), at the sample thickness *t*, is given by the product of two 2D independent functions, the probe *u*(*x*,*y*,*0*) (illumination function) and the object transmission function *T*(*x*,*y*).^23^ It can be shown that the “product approximation” holds if resolution ρ, lateral extension *S* of the illuminating function at the focus and wavelength λ satisfy the following constraint

*b* < ρ *S*/λ, (S8)

which roughly gives the maximum characteristic scale *b* of longitudinal non-uniformity of the object dielectric susceptibility for which the approximation is valid.

In our phase maps ρ is ranging between 40 and 50 nm, the beam size at the focus is *S* ~ 67 nm, λ = 2Å, giving the following limiting thickness: *b* < 13 μm. Consequently, if the whole free standing polymers, which are about 25 µm thick, were characterized by longitudinal variations of the dielectric susceptibility on the whole thickness, this approximation would have not been valid. Conversely, for dilute samples, such as the cases under study, also the product approximation can be considered fulfilled, not limiting the final resolution of the phased maps.

**Resolutions**

In order to evaluate the final resolution of the images, displayed in Fig. 2 and 3 of the manuscript, we adopted the Fourier shell correlation (FSC) criterion. FSC allows to evaluate the correlation in the frequency space of two independent phase maps to determine the resolution threshold at which the two images are best correlated. a broader accepted consistency criterion is to have the FSC of the two images, *g*_1_ and *g*_2_, larger than the so-called half-bit threshold *T*_hb_^4^, the latter being indicated as the black curve in Fig. S5a, 5b, 5c, 5d, for OCT, PS350_thin, PS190 and PS350, respectively. The crossing point, in the frequency domain, between the FSC[*g*_1_,*g*_2_] and *T*_hb_ gives the maximum spatial frequency of the object details contained in the images *g*_1_ and *g*_2_, which corresponds to a spatial resolution ρ. Here, ρ = Δ_image-pixel_ /(*f*/*f*_max_) where *f*_max_ is the Nyquist frequency corresponding to the inverse of the area reconstructed with phasing. The *g*_1_ and *g*_2_ have been obtained by averaging the first two and last three, respectively, of the five available repeated measurements.

***
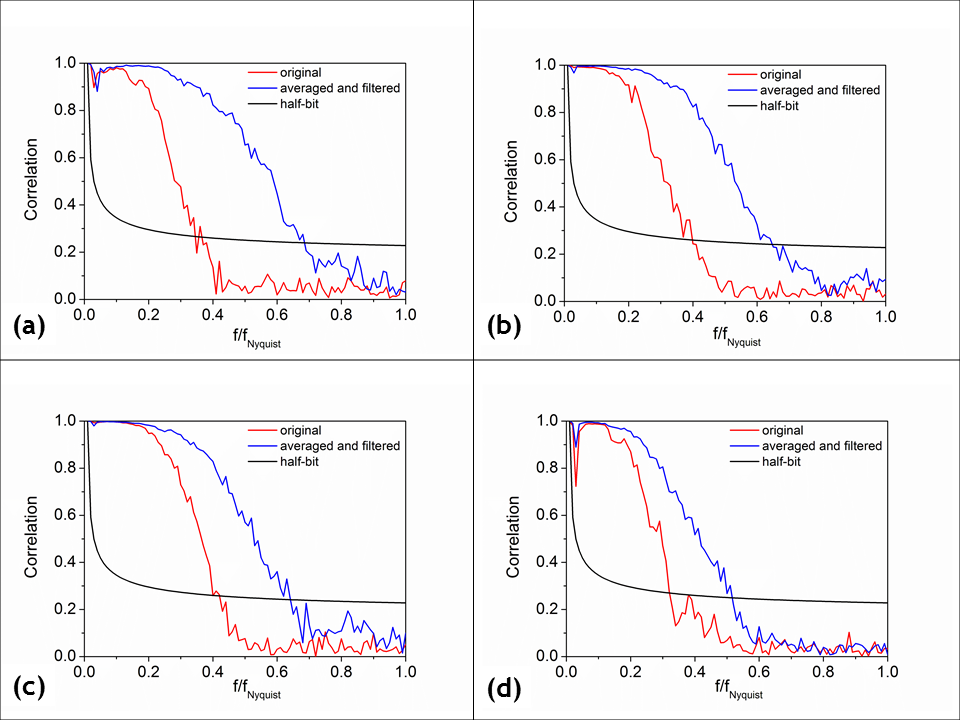
Figure S6.*** *Resolution vs f/f_max_ for the original (red) and averaged / filtered (blue) phased images together with the so-called half-bit threshold T_hb_ reported for samples: OCT (a), PS350_thin (b), PS190 (c) and PS350 (d).*

**Coherence requirements**

Under the experimental conditions of the present experiment, with respect to the longitudinal coherence length, the sample under investigation is actually a thin object.

Indeed the conditions to be fulfilled are:

$L_{Lo}=\frac{\lambda^{2}}{\Delta\lambda}>2d\sin(2\theta_{\max})$. (S9)

$L_{Lo}=\frac{\lambda^{2}}{\Delta\lambda}>4t\sin(\theta_{\max})^{2}$. (S10)

where $\frac{\Delta\lambda}{\lambda}$~ 10^-4^, $\theta_{\max}$= NA = 7.4 mrad, *d*=450 nm and *t*=25 µm.

The longitudinal coherence length ($L_{Lo}$~2 µm) largely exceeds the required values ($2d\sin(2\theta_{\max})$~0.1 µm, $4t\sin(\theta_{\max})^{2}$ ~ 0.005 µm).

As explained in the main text, the coherent illumination of the entire Fresnel Zone plate lens aperture was ensured by using a horizontal aperture close to the source. The focus size at the sample is *d*=450 nm.

For a wavelength λ = 0.2 nm, a sample-detector distance z=2.236 m and a coherently illuminated area *d*=450 nm, being the pixel size of the Pilatus detector Δ_det-pixel_ = 172 µm we have an oversampling ratio $\frac{\lambda z}{2d\Delta_{\det-pix}}$=2.9 suitable to phase retrieval.

**Ptychography scan**

**
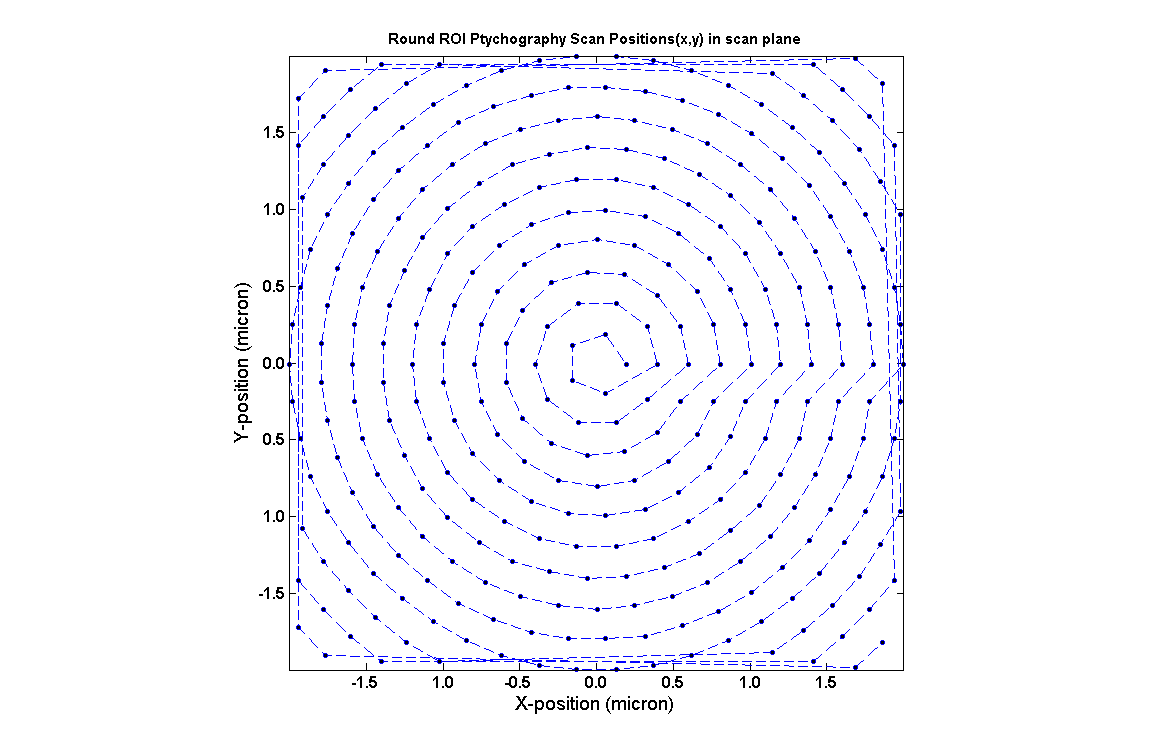
**

***Figure S7.*** *Sketch of the scan acquired in ptychography: concentric circles with a radial step size of 0.2 µm and 5 points in the first circle for a total of 324 scanning points.*

**References and Notes**

^1^Kopylov, Y. V., Popov, A. V. & Vinogradov, A. V. Application of the parabolic wave equation to x-ray diffraction optics, *Optics Commun*. **118**, 619-36 (1995)

^2^ De Caro, L., Cedola, A., Giannini, C., Bukreeva, I. & Lagomarsino, S. In-line phase-contrast imaging for strong absorbing objects, *Phys. Med. Biol*. ***53***, 6619–6637 (2008)

^3^Pogany, A., Gao, D. & Wilkins, S. W. Contrast and resolution in imaging with a microfocus x-ray source, *Rev. Sci. Instrum.* **68**, 2774-2782 (1997)

**^4^** van Heel, M. & Schatz, M. Fourier shell correlation threshold criteria, *J. Struct. Biology* **151**, 250-282 (2005)
